# A diatomic elastic metamaterial for tunable asymmetric wave transmission in multiple frequency bands

**DOI:** 10.1038/s41598-017-05526-3

**Published:** 2017-07-24

**Authors:** Bing Li, Sagr Alamri, K. T. Tan

**Affiliations:** Department of Mechanical Engineering, The University of Akron, Akron, OH 44325-3903 USA

## Abstract

Unidirectional/asymmetric transmission of acoustic/elastic waves has recently been realized by linear structures. Research related to unidirectionality of wave propagation has received intense attention due to potentially transformative and unique wave control applications. However, asymmetric transmission performance in existing devices usually occurs only in a narrow frequency band, and the asymmetric frequencies are always within ultrasound range (above 20 kHz). In this work, we design and propose a linear diatomic elastic metamaterial using dual-resonator concept to obtain large asymmetric elastic wave transmission in multiple low frequency bands. All of these frequency bands can be theoretically predicted to realize one-way wave propagation along different directions of transmission. The mechanisms of multiple asymmetric transmission bands are theoretically investigated and numerically verified by both analytical lattice and continuum models. Dynamic responses of the proposed system in the broadband asymmetric transmission bands are explored and analyzed in time and frequency domains. The effect of damping on the asymmetric wave transmission is further discussed. Excellent agreements between theoretical results and numerical verification are obtained.

## Introduction

Inspired by the remarkable development and extensive application of electrical diode, much effort has been devoted to challenge the one-way propagation of other forms of energy fluxes, such as electromagnetic/optical field^1–3^, thermal flux^4–6^ and solitary wave^7^. Recently, the unidirectional/asymmetric transmission of acoustic/elastic waves has been realized^8, 9^, and related research^10–25^ has become a hot topic by virtue of its various potential applications, such as enhancing medical ultrasound imaging, developing acoustic one-way diode and creating directional de-noise devices.

The pioneer devices of unidirectional/asymmetric acoustic transmission were achieved by utilizing nonlinearity^8–12^. Based on the frequency conversion induced by nonlinear mediums and the filter effect of bandgap phononic crystals, the acoustic time-reversal symmetry is broken, resulting in nonreciprocal wave propagation. Considering the signal distortion by the frequency shift, the poor efficiency of nonlinear conversion and the bulky volume of nonlinear devices, a series of attempts have been made to design linear structures of asymmetric acoustic transmission. One category of linear acoustic rectifiers is the biasing-based linear device^13–15^. Without requiring frequency conversion, a nonreciprocal circulator filled with circulating fluid was presented^13^. The circulating fluid plays the role of an odd-vector biasing, which breaks the acoustic reciprocity by splitting the circulator’s azimuthal resonant modes. To overcome the application challenge in small wavelengths, this approach of fluid-motion-induced biasing was further replaced by an angular-momentum-induced biasing^14, 15^. These biased linear devices can realize asymmetric wave transmission without distorting the wave frequency, but external energy is needed.

Different from nonreciprocal acoustic diodes mentioned above, the other category of one-way device is the linear grating structure^26–32^. Asymmetric wave transmission is generally induced by the specific interactions between incident waves and these passive devices, such as wave diffraction^26–29^, Bragg scattering^30, 31^, mode conversion^30, 32^ and wave refraction^20, 21^ etc. It is worth noting that in all of these passive linear structures, the acoustic reciprocity principle still holds and asymmetric transmission only occurs under specific directions or wave modes. The enhancement performance of asymmetric acoustic transmission has been demonstrated, however, the output signals induced by wave diffraction or refraction are usually difficult to be adjusted and focused due to their split directions and disordered patterns. In addition, because of the inherent wavelength limitation in Bragg scattering and wave diffraction, the asymmetric frequency is always within ultrasound range (above 20 kHz). It is not easy to obtain asymmetric transmission at low frequency range (especially below 1 kHz) by using the small-sized grating structures. Recently, based on surficial localized vibrational modes, a linear diatomic metamaterial with large asymmetric wave transmission is realized^23^. Due to the unique local resonance of metamaterials^33–41^, an extra low asymmetric transmission frequency band, below 1 kHz, can be easily achieved. The asymmetric wave transmission in the passive system can be theoretically predicted and mathematically controlled, without relying on frequency conversion, wave diffraction or external energy.

However, asymmetric wave transmission in these structures is always confined to only one frequency band, which undeniably affect further development and application in broadband situations. In addition, little work has been reported on the quantitative control and mathematical tailoring of various unidirectional transmission bands. In this paper, we propose a linear diatomic acoustic/elastic metamaterial with dual resonator to realize large asymmetric wave transmission in multiple low frequency bands. Remarkably, these asymmetric transmission bands (ATBs) belong to different propagation directions, which are bi-directional tunable. The mechanisms of multiple ATBs are theoretically and mathematically investigated by an analytical mass-in-mass system. Numerical verifications are comprehensively conducted using lattice models and continuum rod models. Transient wave responses in multiple low frequency ATBs are analyzed and evaluated in time and frequency domains. Considering that damping is an intrinsic property of materials, we further investigate the asymmetric wave transmission in a dissipative diatomic system.

## Results

### Design and equivalent models

**Figure 1:**
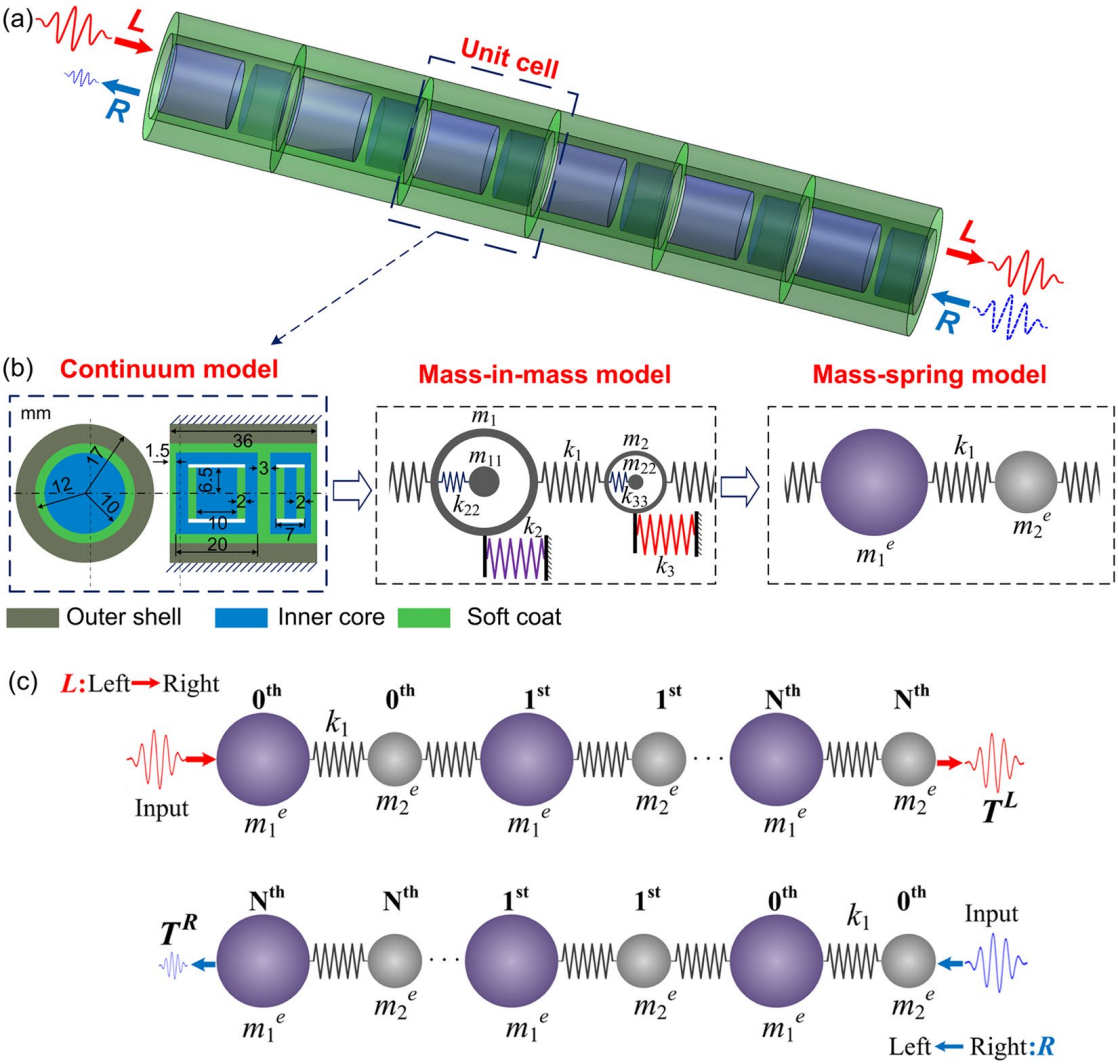
(**a**) Schematic of the diatomic elastic metamaterial with dual-resonator. (**b**) Continuum unit cell and its equivalent mass-in-mass and mass-spring models. (**c**) Directions *L* and *R* in an N-periodic diatomic lattice system with effective “masses”.

The schematic design of the proposed structure is illustrated in Fig. 1(a). The elastic metamaterial consists of several periodical unit cells. Each unit cell is made up of three parts: outer shell, soft coat and inner cores. The outer shell and inner cores are made of relative “hard” materials, such as metallic materials, and the soft coat is made of relative “soft” materials, such as rubber. As shown in Fig. 1(b), each unit cell includes two inner cores with different mass. Each inner core contains one hollow cylinder and an additional cylinder inside the hollow one, which is named as a dual-resonator core. The resonators are connected with each other by the soft materials, and the inner cores are connected with the outer shell also by the soft coat. The outside surface of the outer shell is fixed.

The proposed diatomic unit cell can be analytically described by a mass-in-mass model as illustrated in Fig. 1(b). For the dual-resonator cores, the two outside hollow cylinders can be represented by two outer “masses” with different mass of *m*_1_ and *m*_2_. The two additional solid cylinders inside the hollow cylinders can be enacted by two inner “masses” with mass of *m*_11_ and *m*_22_, respectively. The part with soft material connecting adjacent inner cores can be described by a spring with a stiffness of *k*_1_. The soft coat connecting the outer shell and different inner cores can be enacted by two different springs with stiffnesses of *k*_2_ and *k*_3_. Two additional springs with stiffnesses of *k*_22_ and *k*_33_ are introduced to describe the soft connections between the inner and outer resonators. The heavy outer shell can be approximately considered as a fixed “ground”.

For an infinite lattice system consisting of the mass-in-mass model as shown in Fig. 1(b), the equations of motion for the *j* ^*th*^ unit cell can be written as

1 $$\begin{array}{c}{m}_{1}\frac{{d}^{2}{u}_{1}^{(j)}}{d{t}^{2}}+{k}_{1}(2{u}_{1}^{(j)}-{u}_{2}^{(j)}-{u}_{2}^{(j-1)})+{k}_{2}{u}_{1}^{(j)}+{k}_{22}({u}_{1}^{(j)}-{u}_{3}^{(j)})=0\\ {m}_{2}\frac{{d}^{2}{u}_{2}^{(j)}}{d{t}^{2}}+{k}_{1}(2{u}_{2}^{(j)}-{u}_{1}^{(j)}-{u}_{1}^{(j+1)})+{k}_{3}{u}_{2}^{(j)}+{k}_{33}({u}_{2}^{(j)}-{u}_{4}^{(j)})=0\\ {m}_{11}\frac{{d}^{2}{u}_{3}^{(j)}}{d{t}^{2}}+{k}_{22}({u}_{3}^{(j)}-{u}_{1}^{(j)})=0\\ {m}_{22}\frac{{d}^{2}{u}_{4}^{(j)}}{d{t}^{2}}+{k}_{33}({u}_{4}^{(j)}-{u}_{2}^{(j)})=0\end{array}\}$$

where ${u}_{\alpha }^{(j)}$ *(α* = 1, 2, 3 and 4) are the displacements of mass *m*_1_, *m*_2_, *m*_11_ and *m*_22_ in the *j* ^*th*^ unit cell of the lattice, respectively. For harmonic wave propagation, the displacement for the (*j* + *n*)^*th*^ unit cell is given as

2 $${u}_{\alpha }^{(j+n)}={B}_{\alpha }{e}^{i(\kappa x+nql-\omega t)}$$

where *B*_*α*_ is the amplitude of the displacement, *κ* is the wavenumber, *l* is the distance between two adjacent unit cells and *ω* is the angular frequency^33^. We can obtain the dispersion relations of the proposed system by substituting Eq. (2) into Eq. (1) and setting the determinant equal to zero as

3 $$|\begin{array}{cccc}-{m}_{1}{\omega }^{2}+2{k}_{1}+{k}_{2}+{k}_{22} & -{k}_{1}(1+{e}^{-i\kappa l}) & -{k}_{22} & 0\\ -{k}_{1}(1+{e}^{i\kappa l}) & -{m}_{2}{\omega }^{2}+2{k}_{1}+{k}_{3}+{k}_{33} & 0 & -{k}_{33}\\ -{k}_{22} & 0 & {k}_{22}-{m}_{11}{\omega }^{2} & 0\\ 0 & -{k}_{33} & 0 & {k}_{33}-{m}_{22}{\omega }^{2}\end{array}|=0$$

This mass-in-mass system can be further represented by a simple diatomic mass-spring system (see Fig. 1(b)) with stiffness *k*_1_ and two effective mass ${m}_{1}^{e}$ and ${m}_{2}^{e}$. Compared with the dispersion relation of a monoatomic lattice system, the two effective mass in the equivalent mass-spring model can be derived as

4 $$\begin{array}{c}{m}_{1}^{e}={m}_{1}-\frac{{k}_{2}+{k}_{22}}{{\omega }^{2}}+\frac{{k}_{22}^{2}}{{k}_{22}{\omega }^{2}-{m}_{11}{\omega }^{4}}\\ {m}_{2}^{e}={m}_{2}-\frac{{k}_{3}+{k}_{33}}{{\omega }^{2}}+\frac{{k}_{33}^{2}}{{k}_{33}{\omega }^{2}-{m}_{22}{\omega }^{4}}\end{array}\}$$

In consideration of a finite diatomic lattice system with *N* unit cells (see Fig. 1(c)), we specify two opposite propagation directions. If the wave emitted from the left side first excites the ${m}_{1}^{e}$ end, the direction is denoted by *L*. In contrast, the other direction is denoted by *R*. For direction *L*, the following relations can be readily deduced:

5 $$\begin{array}{c}(2{k}_{1}-{m}_{1}^{e}{\omega }^{2}){\bar{U}}_{q}={k}_{1}({\bar{V}}_{q}+{\bar{V}}_{q-1}),\quad q=1,2,\mathrm{...},N-1\\ (2{k}_{1}-{m}_{2}^{e}{\omega }^{2}){\bar{V}}_{q}={k}_{1}({\bar{U}}_{q}+{\bar{U}}_{q+1}),\quad q=0,1,2,\mathrm{...},N-1\end{array}\}$$

where ${\bar{U}}_{q}$ and ${\bar{V}}_{q}$ are the displacements of ${m}_{1}^{e}$ and ${m}_{2}^{e}$ in the *q* ^*th*^ unit cell. For direction *R*, one can obtain:

6 $$\begin{array}{c}(2{k}_{1}-{m}_{1}^{e}{\omega }^{2}){\bar{U}}_{q}={k}_{1}({\bar{V}}_{q}+{\bar{V}}_{q+1}),\quad q=0,1,2,\mathrm{...},N-1\\ (2{k}_{1}-{m}_{2}^{e}{\omega }^{2}){\bar{V}}_{q}={k}_{1}({\bar{U}}_{q}+{\bar{U}}_{q-1}),\quad q=1,2,\mathrm{...},N-1\end{array}\}$$

According to Eqs (5) and (6), the displacement transmission coefficient between the two adjacent inner unit cells for directions *L* and *R*, ${T}_{q}^{L}$ and ${T}_{q}^{R}$, can be written respectively as

7 $$\begin{array}{c}{T}_{q}^{L}=\frac{{\bar{V}}_{q}}{{\bar{V}}_{q-1}}=\frac{{k}_{1}^{2}}{(2{k}_{1}-{m}_{1}^{e}{\omega }^{2})(2{k}_{1}-{m}_{2}^{e}{\omega }^{2})-{k}_{1}^{2}(2+{T}_{q+1}^{L})},\quad q=1,2,\mathrm{...},N-1\\ {T}_{q}^{R}=\frac{{\bar{U}}_{q}}{{\bar{U}}_{q-1}}=\frac{{k}_{1}^{2}}{(2{k}_{1}-{m}_{1}^{e}{\omega }^{2})(2{k}_{1}-{m}_{2}^{e}{\omega }^{2})-{k}_{1}^{2}(2+{T}_{q+1}^{R})},\quad q=1,2,\mathrm{...},N-1\end{array}\}$$

For direction *L*, the motion equations for the end unit cell (the *N* ^*th*^ unit cell), ${\bar{U}}_{N}$ and ${\bar{V}}_{N}$, can be obtained as

8 $$\begin{array}{c}(2{k}_{1}-{m}_{1}^{e}{\omega }^{2}){\bar{U}}_{N}={k}_{1}({\bar{V}}_{N}+{\bar{V}}_{N-1})\\ ({k}_{1}-{m}_{2}^{e}{\omega }^{2}){\bar{V}}_{N}={k}_{1}{\bar{U}}_{N}\end{array}\}$$

For direction *R*, the motion equations for the end unit cell (${\bar{U}}_{N}$ and ${\bar{V}}_{N}$) can be expressed as

9 $$\begin{array}{c}({k}_{1}-{m}_{1}^{e}{\omega }^{2}){\bar{U}}_{N}={k}_{1}{\bar{V}}_{N}\\ (2{k}_{1}-{m}_{2}^{e}{\omega }^{2}){\bar{V}}_{N}={k}_{1}({\bar{U}}_{N}+{\bar{U}}_{N-1})\end{array}\}$$

Then, for direction *L*, the displacement transmission coefficient of the *N* ^*th*^ unit cell, ${T}_{N}^{L}$, can be derived based on Eq. (8) as

10 $${T}_{N}^{L}=\frac{{k}_{1}^{2}}{(2{k}_{1}-{m}_{1}^{e}{\omega }^{2})({k}_{1}-{m}_{2}^{e}{\omega }^{2})-{k}_{1}^{2}}$$

For direction *R*, according to Eq. (9), a similar displacement transmission coefficient of the *N* ^*th*^ unit cell, ${T}_{N}^{R}$, can be obtained as

11 $${T}_{N}^{R}=\frac{{k}_{1}^{2}}{(2{k}_{1}-{m}_{2}^{e}{\omega }^{2})({k}_{1}-{m}_{1}^{e}{\omega }^{2})-{k}_{1}^{2}}$$

Using the transmission equations of the inner and end unit cells, the displacement transmission coefficients of the entire system for the two opposite directions, *T* ^*L*^ and *T* ^*R*^, can be expressed respectively as

12 $$\begin{array}{c}{T}^{L}=|\frac{{\bar{V}}_{N}}{{\bar{V}}_{0}}|=|(\prod _{q=1}^{N}{T}_{q}^{L})|\\ {T}^{R}=|\frac{{\bar{U}}_{N}}{{\bar{U}}_{0}}|=|(\prod _{q=1}^{N}{T}_{q}^{R})|\end{array}\}$$

### Multiple asymmetric transmission bands

For the proposed continuum unit cell with dual-resonator (see Fig. 1(b)), the materials used for the outer shell and soft coat are aluminum and rubber, respectively. The outer and inner resonators in the inner cores are made of aluminum and lead, respectively. The material properties of all continuum unit cells used in this research are listed in Table 1. The values of the effective “masses” in the mass-in-mass model can be calculated readily by the relevant densities *ρ*_*α*_ and volumes *V*_*α*_ as *m*_*α*_ = *ρ*_*α*_ *V*_*α*_. The approximate values of the effective spring stiffnesses can be estimated by the relevant Young’s modulus *E*_*s*_ and shear modulus *G*_*s*_ of the soft material as

13 $${k}_{\alpha }=\frac{{E}_{s}{A}_{\alpha }}{{l}_{s\alpha }}+\frac{{G}_{s}{A}_{\alpha }}{{l}_{s\alpha }},\quad {G}_{s}=\frac{{E}_{s}}{2(1+{\nu }_{s})}$$

where *A*_*α*_ and *l*_*sα*_ are relevant cross sections and lengths of different sections of the soft connection, *ν*_*s*_ is the Poisson’s ratio of the soft material. By virtue of the complex deformation of soft connections between the adjacent unit cells, it is difficult to ensure the accurate *A*_*α*_ values. Alternatively, more accurate effective spring stiffness are obtained here according to finite element analysis (FEA)^23^. Based on the materials properties, the effective mass and spring stiffness are calculated as *m*_1_ = 1.143 × 10^−2^ *kg*, *m*_2_ = 0.572 × 10^−2^ *kg*, *m*_11_ = 1.505 × 10^−2^ *kg*, *m*_22_ = 0.452 × 10^−2^ *kg*, *k*_1_ = 3.695 × 10^5^ *N/m*, *k*_2_ = 1.785 × 10^5^ *N/m*, *k*_3_ = 0.870 × 10^5^ *N/m*, *k*_22_ = 2.792 × 10^5^ *N/m* and *k*_33_ = 2.792 × 10^5^ *N/m*.

**Table 1:** Material properties in continuum rod models.

Material	H1	H2	H3	H4	S1
	Aluminum	Lead	Al alloy	Steel	Rubber
Young’s modulus (GPa)	70	16	45	210	0.78 × 10^−3^
Density (kg/m^3^)	2770	11340	1800	7850	1200
Poisson’s ratio	0.33	0.45	0.32	0.29	0.47

**Figure 2:**
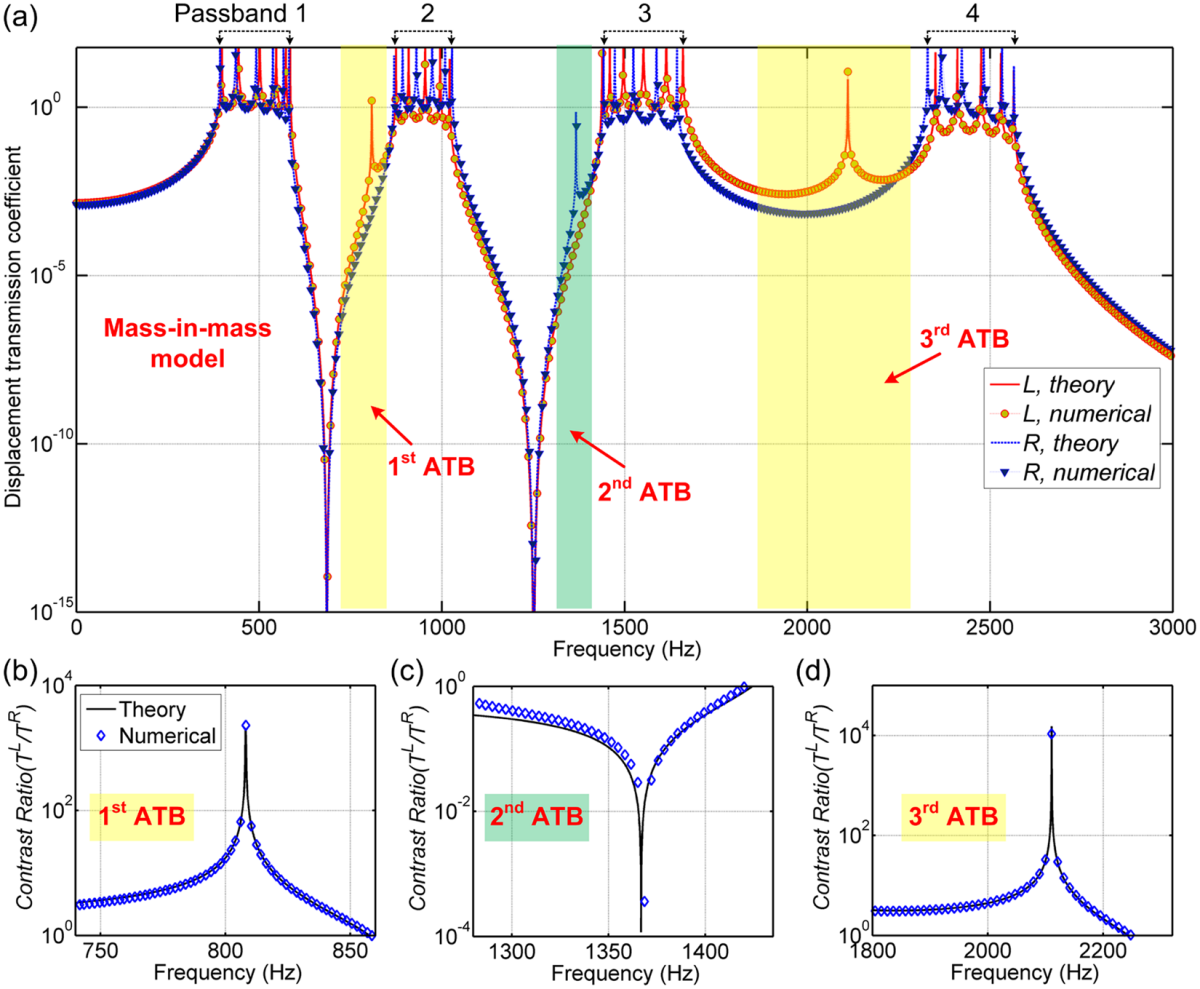
(**a**) Transmission coefficient-frequency profiles obtained by theoretical analysis and FEA. The profiles of asymmetric contrast ratio obtained in the (**b**) 1^st^, (**c**) 2^nd^ and (**d**) 3^rd^ ATBs in the mass-in-mass model.

According to the effective parameters and the Eqs (4), (7) and (10)–(12), the theoretical transmission coefficients along directions *L* and *R* for the proposed structure with 6 unit cells are calculated and displayed in Fig. 2(a). To compare with the theoretical results, a lattice system consisting of 6 mass-in-mass unit cells is built by FEA. A time harmonic displacement at a sweep frequency range is input at one end of the lattice system, while the displacement at the other end is captured as an output. The numerical frequency response functions (FRFs) of the finite mass-in-mass lattice system along the two opposite directions are compared with the theoretical results and shown in Fig. 2(a). It is exhibited that the numerical results agree very well with the theoretical transmission coefficients.

As depicted in Fig. 2(a), there are four passbands for both directions *L* and *R*. The main differences for the two opposite directions occur at the frequency range between two adjacent passbands. For direction *R*, a bandgap with very low transmission coefficient (dip regions shaded by yellow) is obtained between the 1^st^ and 2^nd^ passbands, also between the 3^rd^ and 4^th^ passbands. The wave propagation is significantly attenuated and blocked at these bandgap frequencies. However, for the other direction *L*, there is an interesting peak at either bandgap of *R* direction (see yellow regions in Fig. 2(a)). The transmission coefficients around the two peaks are almost the same as that in passbands. This means that wave can propagate along the direction *L* around these peak frequencies, but prohibited along the direction *R*. On the contrary, at another frequency range between the 2^nd^ and 3^rd^ passbands (regions shaded by green), there is a transmission peak for direction *R*, but a bandgap for direction *L*. It is indicated that the wave can propagate along the direction *R* around this peak frequency, but will be significantly attenuated along the direction *L*. The distinct asymmetric wave transmission shows up in these interesting frequency bands, which are defined as ATBs. More interestingly, these ATBs belong to different wave propagation directions (1^st^ and 3^rd^ ATBs for direction *L*, 2^nd^ ATB for direction *R*). This implies that asymmetric wave propagation can be realized along each direction without requiring any change to the structure, which is extremely beneficial for passive wave control. To quantitatively investigate the asymmetric transmission, an asymmetric contrast ratio of the transmission coefficient along direction *L* to direction *R*, *δ* = *T* ^*L*^/*T* ^*R*^, is introduced. The theoretical and numerical asymmetric contrast ratios in the three ATBs for mass-in-mass models are calculated and displayed in Fig. 2(b), (c) and (d), respectively. Excellent agreements between numerical and theoretical results show that large bidirectional asymmetric wave transmissions (*δ* ≈ 10^4^) in multiple frequency domains are realized in the proposed diatomic elastic metamaterials.

**Figure 3:**
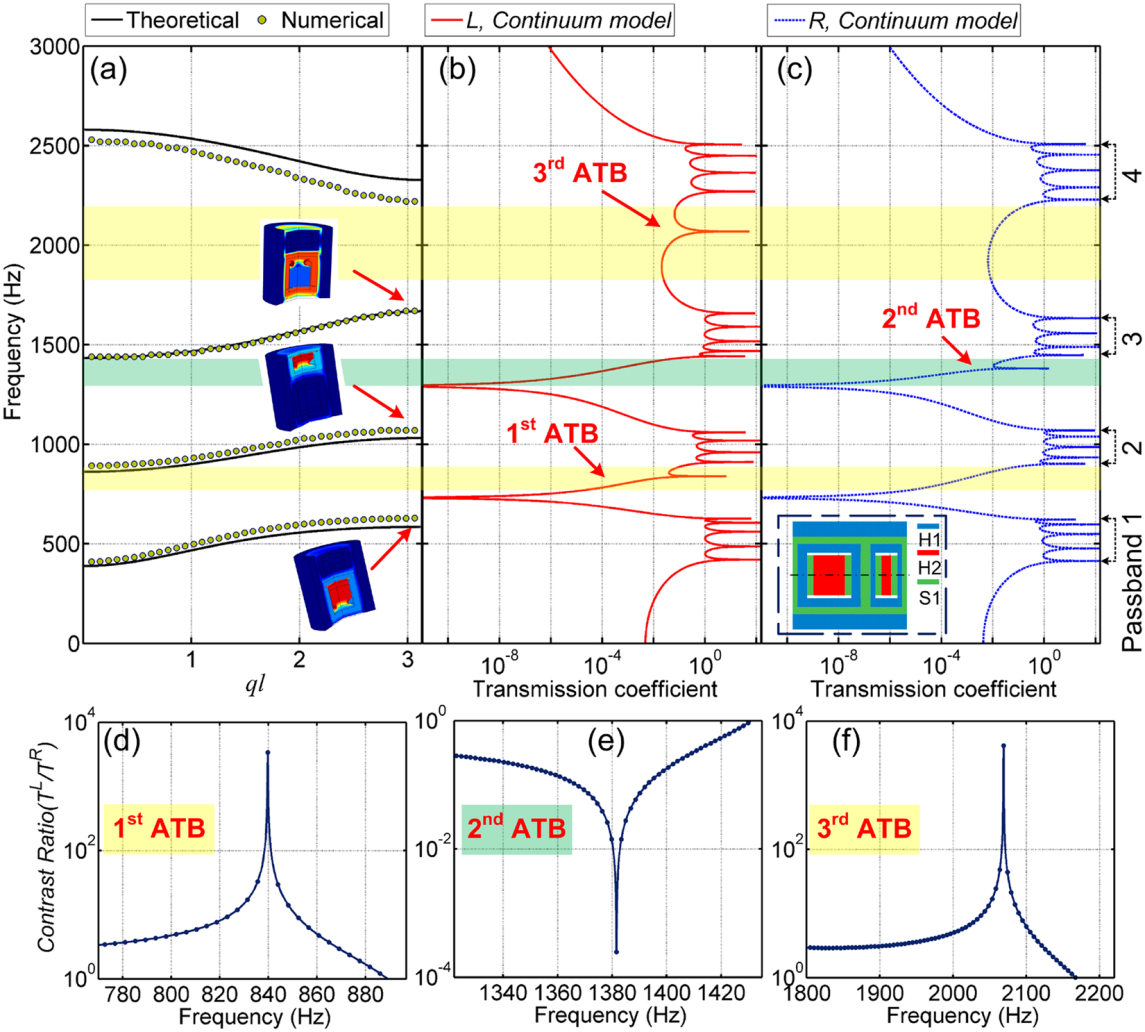
(**a**) Theoretical and numerical dispersion relations of the infinite periodic rod. Numerical FRFs for (**b**) directions *L* and (**c**) *R* for a finite continuum rod consisting of 6 unit cells. Insert in (**c**) shows the periodic unit cell of continuum rod. The profiles of asymmetric contrast ratio obtained in the (**d**) 1^st^, (**e**) 2^nd^ and (**f**) 3^rd^ ATBs in the finite continuum rod model.

In addition to the mass-in-mass lattice system, a more realistic continuum rod model is further built by a commercial FEA software, COMSOL MULTIPHYSICS. The 2D axisymmetric model of the continuum unit cell is depicted in the insert of Fig. 3(c). Based on the Bloch-Floquet theory, the numerical dispersion relations for an infinite continuum rod is obtained and illustrated in Fig. 3(a). The theoretical dispersion relations are calculated by Eq. (3) and also shown in Fig. 3(a), which agree well with the numerical simulation. For a finite continuum rod consisting of 6 unit cells, the numerical FRFs for directions *L* and *R* are plotted in Fig. 3(b) and (c), respectively. Four main passbands exhibited in FRFs agree well with those shown in dispersion spectrums and mass-in-mass model. The theoretical start and end frequencies of these passbands can be calculated by substituting *ql* = 0 or *π* into Eq. (3) as (388.8, 585.6) Hz for 1^st^ passband, (862.1, 1031.8) Hz for 2^nd^ passband, (1434.1, 1668.6) Hz for 3^rd^ passband and (2327.6, 2580.4) Hz for 4^th^ passband. Several resonance modes at the boundary frequencies are captured and visualized in Fig. 3(a), which verify that the bandgaps in the proposed metamaterial are induced by local resonances of various parts of the continuum rod structure.

Comparing the FRFs for the two opposite directions, we can arrive at the same agreement that there exists three ATBs with different wave propagation directions for both the analytical lattice system and continuum model (see shaded regions in Figs 2(a) and 3(b),(c)). Large asymmetric contrast ratios, *δ* of around 10^4^, for the continuum model in the three ATBs are further displayed in Fig. 3(d),(e) and (f).

### Transient responses in time and frequency domain

**Figure 4:**
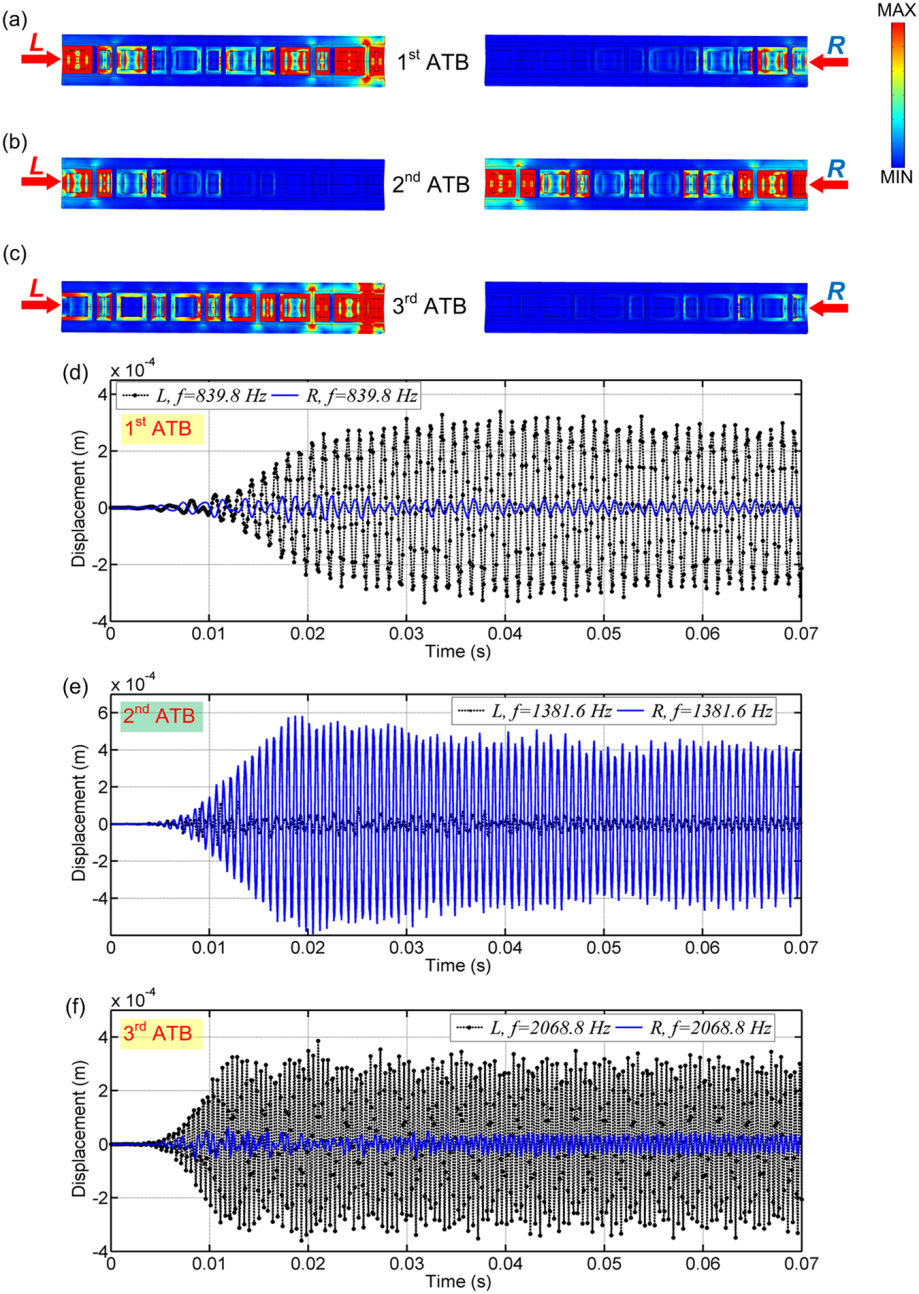
Snapshots of von Mises stress contours captured at the peak frequencies in the (**a**) 1^st^, (**b**) 2^nd^ and (**c**) 3^rd^ ATBs for the structure consisting of 6 unit cells. Displacement-time profiles recorded at the 6^th^ unit cell away from the different input ends under the excitation frequencies of (**d**) 839.8 Hz, (**e**) 1381.6 Hz and (**f**) 2068.8 Hz.

Dynamic response of the proposed structure under a harmonic wave excitation is further analyzed and discussed in this section. For a rod consisting of 6 continuum unit cells, the von Mises stress contours captured at different asymmetric peak frequencies (839.8 Hz, 1381.6 Hz and 2068.8 Hz, see Fig. 3(b) and (c)) are presented in Fig. 4(a),(b) and (c), respectively. It is observed from Fig. 4(a) and (c) that in the 1^st^ and 3^rd^ ATBs, significant wave transmission at the output end is obtained when wave propagates from *L* direction. However, when wave propagates from *R* direction, wave transmission is blocked around the input end and the transmitted wave is extremely weak. On the contrary, Fig. 4(b) shows that in the 2^nd^ ATB, the wave propagation direction is reverse. Large wave is observed at the output end when wave propagates along direction *R*, but attenuated significantly along direction *L*, which agrees very well with the theoretical and numerical transmission coefficient profiles (see Figs 2 and 3).

To investigate the transient response in time domain, three prescribed displacements with different peak frequencies (839.8 Hz, 1381.6 Hz and 2068.8 Hz) are input to a continuum rod consisting of 100 unit cells. Under different excitation frequencies, the displacement-time profiles recorded at the 6^th^ unit cell away from different input ends are compared in Fig. 4(d),(e) and (f), respectively. For the same propagation distance, in the 1^st^ and 3^rd^ ATBs, elastic wave transmission along direction *L* is significantly larger than that along direction *R*; while in the 2^nd^ ATB, the transmitted waves from direction *R* is much larger than that from direction *L*. It is further demonstrated that asymmetric wave transmission in multiple frequency bands can be achieved in the proposed structure, and the one-way transmission is bilaterally controllable.

**Figure 5:**
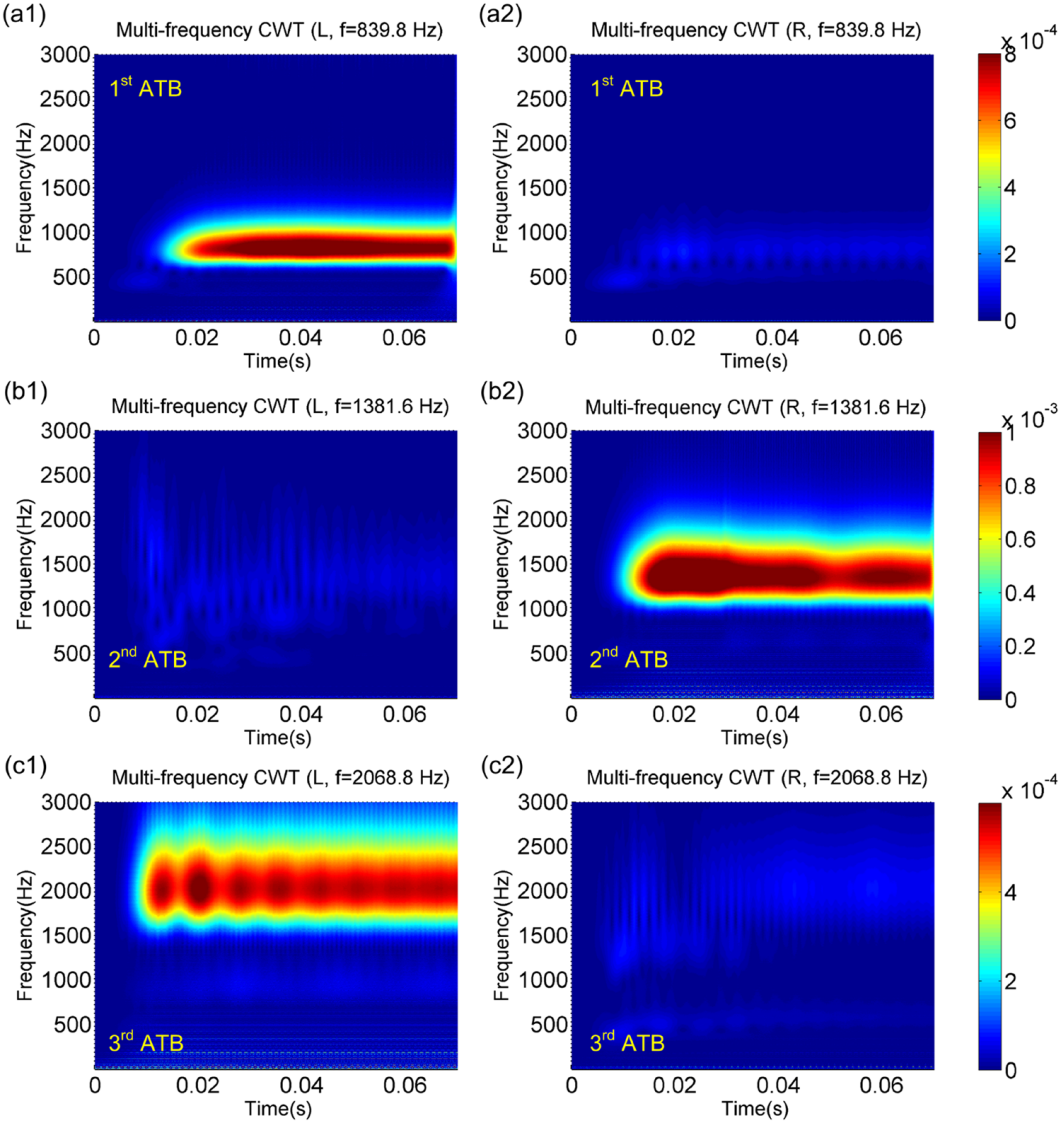
Multi-frequency CWT results of the displacements obtained at the 6^th^ unit cell far from different input ends under the peak excitation frequencies in the (**a**) 1^st^, (**b**) 2^nd^ and (**c**) 3^rd^ ATBs.

To analyze the frequency content of the response signals, a continuous wavelet transformation (CWT) is conducted to process the output displacements due to its local and self-adaptive time-frequency properties^42^. By virtue of the high time resolution, a Gabor wavelet is selected in this research as the mother wavelet function. The comparisons of the multi-frequency CWT results of the output signals under different excitation frequencies are illustrated in Fig. 5(a),(b) and (c). It is clear that for each ATB, the main frequency ranges of the output signals are all around the excitation frequencies. This implies that no frequency conversion occurs in the asymmetric wave transmission of the proposed structure, and the output signal is not distorted. It is also obvious that in the 1^st^ and 3^rd^ ATBs, the wave transmission along *L* direction (see Fig. 5(a1) and (c1)) is far greater than that along *R* direction (see Fig. 5(a2) and (c2)). In the 2^nd^ ATB, the output signal from the direction *R* (see Fig. 5(b2)) is much stronger than that from the direction *L* (see Fig. 5(b1)). Large bidirectional asymmetric wave transmission in multiple frequency bands is verified in both time and frequency domains.

### Tunable asymmetric transmission

Based on above sections, there is a peak transmission coefficient in each ATB for all cases, which leads to the asymmetric transmission. The peak frequencies can be mathematically expounded and controlled by the displacement transmission equations. It is noted from Eqs (10) and (11) that the main difference of the displacement transmission coefficients along the two directions is the transmission equation at the *N* ^*th*^ unit cell, i.e. ${T}_{N}^{L}$ and ${T}_{N}^{R}$. ${T}_{N}^{L}$ and ${T}_{N}^{R}$ will be infinite at several unique frequencies, at which their denominators become zero. According to Eqs (10) and (11), we can obtain that for direction *L*, ${T}_{N}^{L}$ becomes infinite at the peak frequencies of *ω*_*L*1_, *ω*_*L*2_, *ω*_*L*3_ and *ω*_*L*4_, where

14 $${\omega }_{L1}=522.6\,{\rm{{\rm H}}}{\rm{z}},\,{\omega }_{L2}=823.6\,{\rm{{\rm H}}}{\rm{z}}{\boldsymbol{,}}\,{\omega }_{L3}=1545.2\,{\rm{{\rm H}}}{\rm{z}}{\boldsymbol{,}}\,{\omega }_{L4}=2146.4\,{\rm{{\rm H}}}{\rm{z}}$$

For direction *R*, ${T}_{N}^{R}$ becomes infinite at the peak frequencies of *ω*_*R*1_, *ω*_*R*2_, *ω*_*R*3_ and *ω*_*R*4_, where

15 $${\omega }_{R1}=475.2\,{\rm{{\rm H}}}{\rm{z}}{\boldsymbol{,}}\,{\omega }_{R2}=967.8\,{\rm{{\rm H}}}{\rm{z}}{\boldsymbol{,}}\,{\omega }_{R3}=1374.2\,{\rm{{\rm H}}}{\rm{z}}{\boldsymbol{,}}\,{\omega }_{R4}=2390.3\,{\rm{{\rm H}}}{\rm{z}}$$

It is depicted in Figs 2 and 3 that for direction *L*, the frequencies *ω*_*L*1_ and *ω*_*L*3_ belong to the passbands ((388.8, 585.6) Hz and (1431.4, 1668.6) Hz), but the frequencies *ω*_*L*2_ and *ω*_*L*4_ are within the bandgaps ((585.6, 862.1) Hz and (1668.6, 2327.6) Hz). For direction *R*, only *ω*_*R*3_ is within the bandgaps ((1031.8, 1434.1) Hz), the other three peak frequencies belong to the passbands. There is no effect on the transmission characteristics when the peak frequencies fall outside the bandgaps. But when the peak frequencies are within the bandgaps (*ω*_*L*2_, *ω*_*L*4_ and *ω*_*R*3_), asymmetric wave transmission occurs (see Figs 2 and 3).

**Figure 6:**
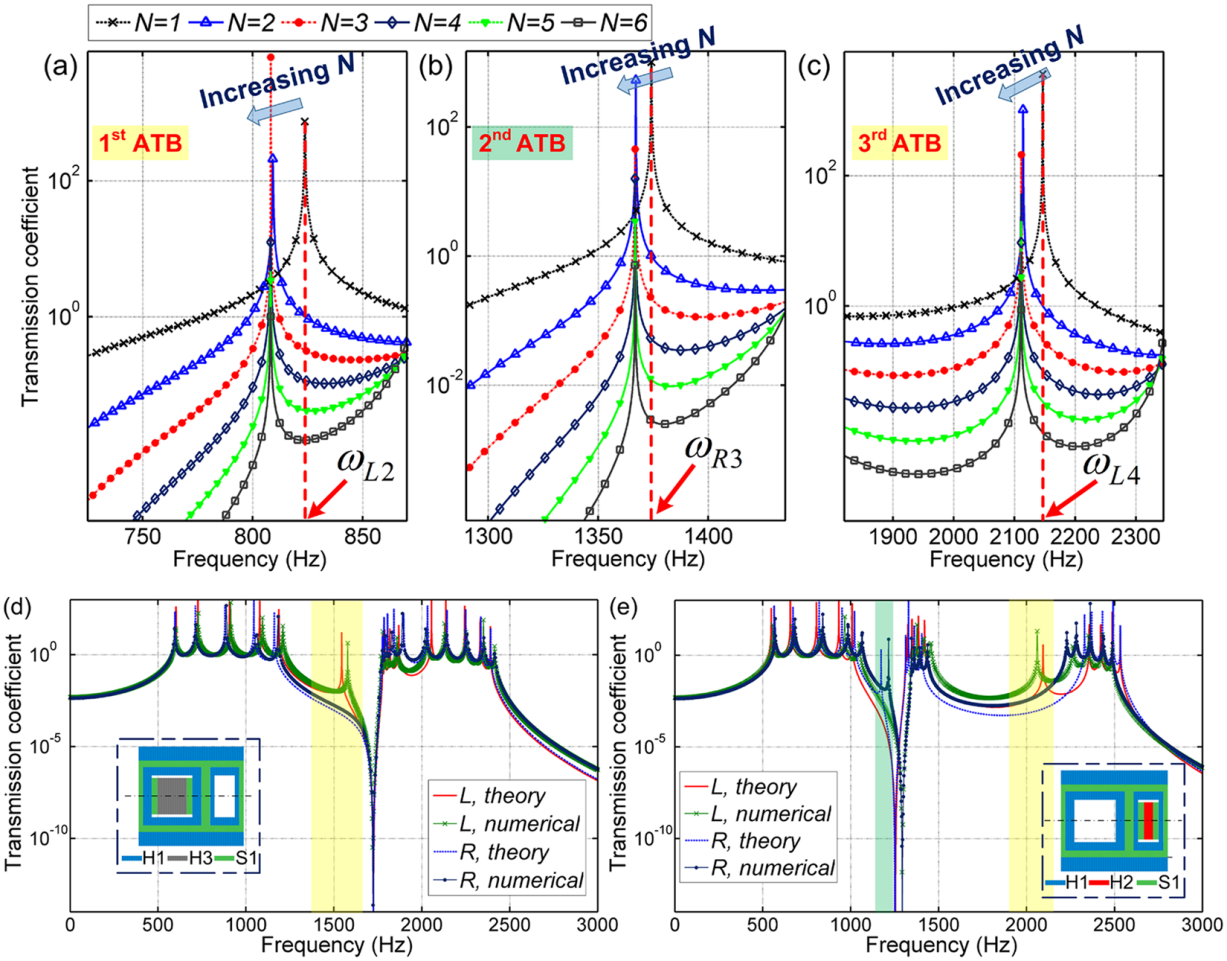
Effect of the unit cell number on the asymmetric peak frequencies in the (**a**) 1^st^, (**b**) 2^nd^ and (**c**) 3^rd^ ATBs. Theoretical and numerical transmission coefficient-frequency profiles for a finite continuum rod built by the unit cell with (**d**) one ATB and (**e**) two ATBs.

Considering the coupling between the end and inner unit cells (see Eq. (7)), the effect of the total number of unit cells on the three asymmetric peak frequencies is further illustrated in Fig. 6(a),(b) and (c), respectively. It is obtained that the peak frequencies are exactly equal to *ω*_*L*2_, *ω*_*R*3_ or *ω*_*L*4_, when there is only one unit cell. The peak frequencies slightly decrease with increase in the number of unit cells, but converge when there are more than three unit cells. The effect of unit cell number on the asymmetric peak frequencies is thus considered negligible, since it will not change the ATBs.

Therefore, the bidirectional ATBs can be theoretically predicted and mathematically controlled. The theoretical frequency ranges of the passbands and bandgaps can be calculated by the equation of dispersion relations, Eq. (3). The peak frequencies can be estimated by Eqs (10) and (11). We can design the sizes and choose the materials of the unit cell to guide the peak frequencies to fall outside or within the passbands, thereby tailoring the one-way transmission bands. Two other designs of continuum unit cells, as depicted in the insert of Fig. 6(d) and (e), are supplemented to verify the reliability of the tunable asymmetric transmission. Various ATBs are obtained in the relevant transmission coefficient profiles. Theoretical analysis agrees very well with numerical verification for each case. The tunable properties and tailoring of various ATBs could pose significant potential in further development and application of transformative unidirectional devices.

## Discussion

In this work, multiple tunable ATBs in the proposed finite structure have been comprehensively investigated and verified by analytical model and numerical simulation. In physics, we can use a concept of surficial localized vibrational mode to discuss the unique phenomena, which is initially found in semiconductor superlattices^43, 44^. For a finite or semi-infinite superlattice, within the bandgaps, several localized vibrational modes can be induced at the free surface or a defect layer due to the resonance interaction of incident pulse and the inhomogeneity. By changing the stacking sequence of two kinds of materials, the surficial localized modes will occur or disappear. But in an infinite superlattice, it is not applicable. When we compare the asymmetric peak frequencies with the surficial localized modes, we can find several similar characteristics. As shown in Figs 2 and 3, various peak frequencies in FRFs denote various vibration modes. For a continuum rod consisting of infinite unit cell proposed in this research, no singularity shows up within the bandgaps (see Fig. 3(a)). However, in the finite periodical structure, several vibration modes captured at the end surfaces have a shift to the bandgaps, leading to asymmetric transmission (see Fig. 3(b) and (c)). Although the bandgap mechanisms for the proposed metamaterials and the semiconductor superlattice are totally different, the peak frequencies around the end unit cells or free surfaces are analogous.

**Figure 7:**
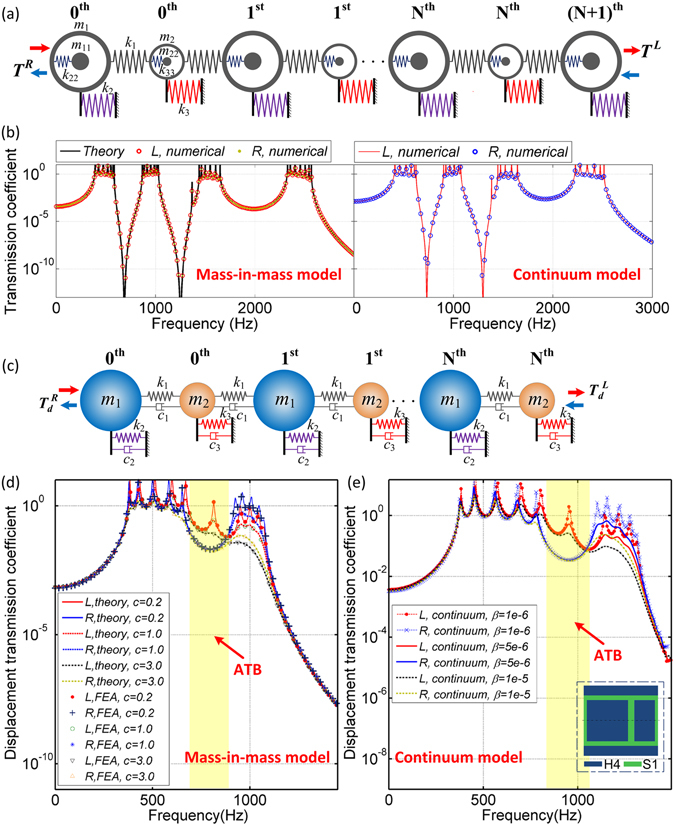
(**a**) Symmetric diatomic lattice model and (**b**) relevant theoretical and numerical transmission coefficient-frequency profiles along directions *L* and *R*. (**c**) Dissipative diatomic lattice system. (**d**) Effect of damping on ATBs in the dissipative lattice and (**e**) continuum models.

In mechanisms, the different surficial vibrational modes along directions *L* and *R* originate from the asymmetric boundary conditions of the two end unit cells. To illustrate this point, we further build a finite symmetric lattice model, as shown in Fig. 7(a), which consists of (N + 1) unit cells of ${m}_{1}^{e}$ and N unit cells of ${m}_{2}^{e}$. The end unit cells for two opposite directions are both ${m}_{1}^{e}$. The transmission coefficient between the two adjacent inner unit cells in this symmetric system can be calculated as

16 $${T}_{q}^{L}={T}_{q}^{R}=\frac{{k}_{1}^{2}}{(2{k}_{1}-{m}_{1}^{e}{\omega }^{2})(2{k}_{1}-{m}_{2}^{e}{\omega }^{2})-{k}_{1}^{2}(2+{T}_{q+1}^{L})},\quad q=1,2,\mathrm{...},N$$

The motion equations for the two end unit cells along directions *L* and *R* should be same and can be written as

17 $$\begin{array}{c}(2{k}_{1}-{m}_{2}^{e}{\omega }^{2}){\bar{V}}_{N}={k}_{1}({\bar{U}}_{N}+{\bar{U}}_{N+1})\\ ({k}_{1}-{m}_{1}^{e}{\omega }^{2}){\bar{U}}_{N+1}={k}_{1}{\bar{V}}_{N}\end{array}\}$$

Then the displacement transmission coefficients of the end unit cells are derived as

18 $${T}_{N+1}^{L}={T}_{N+1}^{R}=\frac{{k}_{1}^{2}}{(2{k}_{1}-{m}_{2}^{e}{\omega }^{2})({k}_{1}-{m}_{1}^{e}{\omega }^{2})-{k}_{1}^{2}}$$

The displacement transmission coefficients of the entire symmetric system are further obtained as

19 $${T}^{L}={T}^{R}=|(\prod _{q=1}^{N+1}{T}_{q}^{L})|$$

For a symmetric structure with 7 unit cells of ${m}_{1}^{e}$ and 6 unit cells of ${m}_{2}^{e}$, the theoretical transmission coefficients along directions *L* and *R* are calculated and displayed in Fig. 7(b). The relevant numerical verifications obtained by both the mass-in-mass and continuum models are compared. It is illustrated that excellent agreements between numerical and theoretical results are obtained and there are no ATBs in the symmetric systems. It’s totally symmetric between the transmission characteristics along directions *L* and *R*.

In addition, because damping is always an intrinsic property of materials, we further discuss the effect of damping on the ATBs, which should be potentially important for experimental testing. A dissipative diatomic model is proposed and depicted in Fig. 7(c), where three different damping elements are introduced. *c*_1_, *c*_2_ and *c*_3_ are the damping coefficients introduced alongside the spring stiffness of *k*_1_, *k*_2_ and *k*_3_, respectively. For this dissipative lattice system, the effective mass ${m}_{1}^{e}$ and ${m}_{2}^{e}$ for the diatomic unit cells can be derived respectively as

20 $${m}_{1}^{e}={m}_{1}-\frac{{k}_{2}}{{\omega }^{2}}+\frac{i{c}_{2}}{\omega },\quad {m}_{2}^{e}={m}_{2}-\frac{{k}_{3}}{{\omega }^{2}}+\frac{i{c}_{3}}{\omega }$$

According to the motion equations, the following relations can be obtained for direction *L*,

21 $$\begin{array}{c}(2{k}_{1}-{m}_{1}^{e}{\omega }^{2}-2i\omega {c}_{1}){\bar{U}}_{q}=({k}_{1}-i\omega {c}_{1})({\bar{V}}_{q}+{\bar{V}}_{q-1}),\quad q=1,2,\mathrm{...},N-1\\ (2{k}_{1}-{m}_{2}^{e}{\omega }^{2}-2i\omega {c}_{1}){\bar{V}}_{q}=({k}_{1}-i\omega {c}_{1})({\bar{U}}_{q}+{\bar{U}}_{q+1}),\quad q=0,1,2,\mathrm{...},N-1\end{array}\}$$

Similarly, for direction *R*, we have

22 $$\begin{array}{c}(2{k}_{1}-{m}_{1}^{e}{\omega }^{2}-2i\omega {c}_{1}){\bar{U}}_{q}=({k}_{1}-i\omega {c}_{1})({\bar{V}}_{q}+{\bar{V}}_{q+1}),\quad q=0,1,2,\mathrm{...},N-1\\ (2{k}_{1}-{m}_{2}^{e}{\omega }^{2}-2i\omega {c}_{1}){\bar{V}}_{q}=({k}_{1}-i\omega {c}_{1})({\bar{U}}_{q}+{\bar{U}}_{q-1}),\quad q=1,2,\mathrm{...},N-1\end{array}\}$$

On basis of Eqs (21) and (22), the displacement transmission coefficient between the two adjacent inner unit cells for directions *L* and *R* can be calculated respectively as

23 $$\begin{array}{c}{T}_{q}^{L}=\frac{{({k}_{1}-i\omega {c}_{1})}^{2}}{(2{k}_{1}-{m}_{1}^{e}{\omega }^{2}-2i\omega {c}_{1})(2{k}_{1}-{m}_{2}^{e}{\omega }^{2}-2i\omega {c}_{1})-{({k}_{1}-i\omega {c}_{1})}^{2}(2+{T}_{q+1}^{L})},\quad q=1,2,\mathrm{...},N-1\\ {T}_{q}^{R}=\frac{{({k}_{1}-i\omega {c}_{1})}^{2}}{(2{k}_{1}-{m}_{1}^{e}{\omega }^{2}-2i\omega {c}_{1})(2{k}_{1}-{m}_{2}^{e}{\omega }^{2}-2i\omega {c}_{1})-{({k}_{1}-i\omega {c}_{1})}^{2}(2+{T}_{q+1}^{R})},\quad q=1,2,\mathrm{...},N-1\end{array}\}$$

For direction *L*, the motion equations for the end unit cell in this dissipative system are written as

24 $$\begin{array}{c}(2{k}_{1}-{m}_{1}^{e}{\omega }^{2}-2i\omega {c}_{1}){\bar{U}}_{N}=({k}_{1}-i\omega {c}_{1})({\bar{V}}_{N}+{\bar{V}}_{N-1})\\ ({k}_{1}-{m}_{2}^{e}{\omega }^{2}-i\omega {c}_{1}){\bar{V}}_{N}=({k}_{1}-i\omega {c}_{1}){\bar{U}}_{N}\end{array}\}$$

For direction *R*,

25 $$\begin{array}{c}({k}_{1}-{m}_{1}^{e}{\omega }^{2}-i\omega {c}_{1}){\bar{U}}_{N}=({k}_{1}-i\omega {c}_{1}){\bar{V}}_{N}\\ (2{k}_{1}-{m}_{2}^{e}{\omega }^{2}-2i\omega {c}_{1}){\bar{V}}_{N}=({k}_{1}-i\omega {c}_{1})({\bar{U}}_{N}+{\bar{U}}_{N-1})\end{array}\}$$

The displacement transmission coefficient of the *N* ^*th*^ unit cell along different directions can be obtained as

26 $$\begin{array}{c}{T}_{N}^{L}=\frac{{({k}_{1}-i\omega {c}_{1})}^{2}}{(2{k}_{1}-{m}_{1}^{e}{\omega }^{2}-2i\omega {c}_{1})({k}_{1}-{m}_{2}^{e}{\omega }^{2}-i\omega {c}_{1})-{({k}_{1}-i\omega {c}_{1})}^{2}}\\ {T}_{N}^{R}=\frac{{({k}_{1}-i\omega {c}_{1})}^{2}}{(2{k}_{1}-{m}_{2}^{e}{\omega }^{2}-2i\omega {c}_{1})({k}_{1}-{m}_{1}^{e}{\omega }^{2}-i\omega {c}_{1})-{({k}_{1}-i\omega {c}_{1})}^{2}}\end{array}\}$$

According to Eqs (12), (20), (23) and (26), the theoretical displacement transmission coefficients along directions *L* and *R* (${T}_{d}^{L}$ and ${T}_{d}^{R}$) for a dissipative lattice system with 6 unit cells are evaluated and compared in Fig. 7(d). The mass and spring stiffness utilized in this dissipative system are *m*_1_ = 0.030 *kg*, *m*_2_ = 0.015 *kg*, *k*_1_ = 2.0 × 10^5^ *N/m*, *k*_2_ = 1.5 × 10^5^ *N/m* and *k*_3_ = 1.0 × 10^5^ *N/m*. The damping coefficients of *c*_1_, *c*_2_ and *c*_3_ are all taken as *c*. Three different values (0.2 Ns/m, 1.0 Ns/m and 3.0 Ns/m) are selected respectively for *c* to investigate the effect of damping on the asymmetric wave transmission. The corresponding numerical verifications obtained by the mass-in-mass model are displayed in Fig. 7(d), which agree very well with the analytical model. Three different Rayleigh damping coefficients (*β* = 1.0 × 10^−6^, 0.5 × 10^−5^ and 1.0 × 10^−5^) are applied to soft material (S1) in the dissipative continuum model (see the insert of Fig. 7(e)), respectively. The numerical transmission coefficients along direction *L* and *R* obtained by the dissipative continuum model are shown in Fig. 7(e). The other material properties for the continuum model are listed in Table 1. It is clearly observed from both lattice and continuum models that the ATBs in the proposed dissipative diatomic metamaterials still exist even when the damping coefficient is relative high. Therefore, we believe that it is feasible to perform experimental verifications on the asymmetric wave transmission in the near future. We can adjust the electro-dynamic shaker to generate the input excitation, and use the scanning laser Doppler vibrometer to capture the output displacement at a sweep frequency range, then calculate the transmission coefficients. It is expected that experimental testing agrees well with theoretical and numerical results.

To conclude, we have designed a linear diatomic elastic metamaterial with dual-resonator to realize large asymmetric elastic wave transmission in multiple low frequency bands. We have presented systematic theoretical analysis and numerical verification to investigate the one-way transmission in the proposed structure. Excellent agreements between theoretical results and numerical simulations are obtained. Asymmetric transmission shows up within multiple very low frequency domains, which is induced by the asymmetric boundary condition and the self-coupling of dual resonators, without relying on frequency conversion or external energy. This inevitably preserves the incident wave frequency and propagation direction. Remarkably, the multiple ATBs are revealed to belong to different propagation directions, which can be theoretically predicted and mathematically tailored to realize one-way transmission along various designed routes. In addition, it is verified that the unique asymmetric transmissions still exist in the proposed dissipative diatomic system, which provides theoretical support for further experimental verifications. This easy bandgap tunability and bidirectional tailorability could pose tremendous beneficial potential for acoustic/elastic wave rectifiers and transmission control.

## Methods

### Finite element models

All the numerical continuum rod models presented in this article were built by a commercial FEA software, COMSOL MULTIPHYSICS. 2D axisymmetric model in solid mechanics module was utilized to investigate the dynamic responses of the proposed structure. The numerical dispersion relations shown in Fig. 3(a) were computed using eigenfrequency and parameter sweep study in one unit cell. The periodic condition of Floquet periodicity was applied to the boundaries of the unit cell. We conducted the frequency domain study to obtain the FRFs of the finite diatomic metamaterials and plotted in Figs 3 and 7. The displacement transmission coefficients along different directions were calculated at a sweep frequency range. The transient responses illustrated in Fig. 4(d),(e) and (f) were calculated using the time dependent study. The excitation signals are prescribed harmonic displacements with the amplitude 2 × 10^−4^ m and different peak frequencies. To avoid the wave reflection from the end edges, 100 unit cells were built for the transient responses. The sampling frequency is 10^4^ Hz.

### Continuous wavelet transformation

For a square-integrable signal *f* (*t*), the CWT definition in time and frequency domains is described, respectively, as

27 $$Wf(a,b)=\frac{1}{\sqrt{a}}{\int }_{-\infty }^{+\infty }f(t)\bar{\psi }(\frac{t-b}{a})dt$$

and

28 $$Wf(a,b)=\frac{\sqrt{a}}{2\pi }{\int }_{-\infty }^{+\infty }\hat{f}(\omega )\overline{\hat{\psi }(a\omega )}\,{\rm{e}}{\rm{x}}{\rm{p}}(i\omega b){\rm{d}}\omega $$

where *a* and *b* are the scale and translation factors, *i* and *ω* are the imaginary number and circular frequency, respectively. The mother wavelet function in CWT is described by *ψ*(*t*), which should satisfy

29 $${\int }_{-\infty }^{+\infty }\frac{{|\psi (\omega )|}^{2}}{\omega }d\omega  < \infty $$

We used a Gabor wavelet as the mother wavelet function in this research because of its high time resolution^42^, which is expressed as

30 $${\psi }_{G}\,(t)=\frac{1}{\sqrt[4]{\pi }}\sqrt{\frac{{\omega }_{0}}{\gamma }}\,{\rm{e}}{\rm{x}}{\rm{p}}[-\frac{({\omega }_{0}/\gamma )}{2}{t}^{2}]\,{\rm{e}}{\rm{x}}{\rm{p}}(i{\omega }_{0}t)$$
